# Editorial: Insights in heart failure and transplantation: 2023

**DOI:** 10.3389/fcvm.2025.1619797

**Published:** 2025-05-19

**Authors:** Andrea Stefanini, Michael Henein, Nicolò Ghionzoli, Matteo Cameli

**Affiliations:** ^1^ Department of Medical Biotechnologies, Division of Cardiology, University of Siena, Siena, Italy; ^2^ Department of Public Health and Clinical Medicine, Umeå University, Umeå, Sweden; ^3^ Imperial College London, London, United Kingdom

**Keywords:** advanced heart failure, heart transplantation, left ventricular assist device, novel strategies, cardiac replacement therapy

**Editorial on the Research Topic**
Insights in heart failure and transplantation: 2023

In this editorial we present the contributing articles of the Research Topic “*Insights in Heart Failure and Transplantation: 2023*” of the journal Frontiers in Cardiovascular Medicine.

## Optimizing strategies in advanced HF

Advanced stages of heart failure (HF) are marked by persistence of severe symptoms despite optimal medical and device therapy, severe impairment of exercise capacity (with inability to exercise or peak oxygen consumption <12 ml/kg/min or <50% predicted value) and frequent hospitalizations. The progression to advanced HF is characterized, in most cases, by the predominant concept of exhaustion of neurohormonal therapeutics, in which medical therapy is no longer able to counteract the pathological mechanisms typical of HF (sympathetic and renin-angiotensin-aldosterone system hyperactivation, fluid retention) (https://doi.org/10.1016/j.jchf.2023.05.013). Thus, early detection of this transition is key to prompt referral and evaluation of advanced HF treatment options. In this context, hemodynamic assessment (e.g., through pulmonary capillary wedge pressure >15 mmHg or cardiac index <2.2, L/min/m^2^), is crucial for risk stratification in HF, even if limited by thresholds for each parameter which are occasionally arbitrary. A novel hemodynamic variable, the Myocardial Performance Score (MPS), was recently introduced by Grinstein, as an objective assessment of the energetic state of patients. It was calculated as (aortic pulsatility index × cardiac power output)/2 and it was found that at an MPS of <0.5, potential energy rises, while efficiency decreases sharply, resulting in a reduction of further power output from the heart. Hence, this parameter, also validated in a cohort of 224 Stage D HF patients, is proposed as a patient-specific threshold for decompensation or recovery, defining the turning point for patients with advanced HF.

Upon determination of the optimal timing for referral to advanced therapies, numerous novel devices are emerging (but not yet recommended) as therapeutic options: these include baroreflex activation therapy, vagus nerve stimulation and cardiac contractility modulation, based on their specific advantages in targeting the underlying pathophysiological or anatomical abnormalities in HF. Balgobind et al. summarize these innovations (comprising transcatheter interatrial shunt creation and transcatheter valve repair systems) and postulated the existence of four distinct HF phenoprofiles based on response to guideline-directed medical therapy and eligibility for heart replacement therapy (HRT), to better tailor emerging technologies to appropriate patient groups.

At any rate, discussion in multidisciplinary Heart Teams, which should include HF experts as well as interventional cardiologists and cardiac surgeons, proves to be of crucial importance to prevent futile interventional procedures, determine the optimal timing for advanced therapies, and ensure a strong HF network between spoke and hub, in order to avoid late referrals that could preclude future eligibility for HRT (Valente et al.).

## Predicting outcomes in advanced HF

Prognosis and outcomes in advanced HF patients are shaped by the application of classical predictors of death and re-hospitalization, such as N-terminal pro-brain natriuretic peptide or troponin. Besides, secretoneurin (SN) is a neuropeptide that acts through the inhibition of calmodulin-dependent kinase II, influencing calcium handling; it is emerging as a novel biomarker capable of stratifying and predicting mortality risk in patients with acute and chronic HF. Plášek et al. showed that plasma SN levels are significantly higher in patients with dilated cardiomyopathy than in patients with ischemic cardiomyopathy, with 88% sensitivity and 61% specificity and a cut-off value 13.3 pmol/L. These findings could reflect a more advanced molecular stage of HF and/or dysfunction in calcium handling, potentially improving the diagnostic and therapeutic pathways in advanced HF.

Machine learning (ML) could also be a valuable resource which simplifies routine clinical data into reproducible models and may yield new insights that could support more personalized care, also in advanced HF. Recently, Mardini et al. created a ML model which identifies the clinical features that significantly impact outcomes in heart transplantation (HTx) candidates (supported with either Impella or Intra-aortic Balloon Pump), enabling intensified monitoring, optimization, and care escalation selectively in these patients.

## Advances in HTx and LVAD

Although HTx remains the gold standard treatment for selected patients with end-stage HF, this is limited by several technical and planning factors (ISHLT guidelines, https://doi.org/10.1016/j.healun.2024.05.010) and the burden of advanced HF far exceeds the availability of donor organs. In order to improve donor heart utilization and post-HTx outcomes, Masroor et al. analyzed 361 patients and demonstrated that the donor/recipient ascending aortic diameter ratio could be a novel potential metric for donor selection. A ratio >0.8032 may contribute to better survival and fewer postoperative complications, resulting in a shorter ventilation and surgery time, less use of extracorporeal membrane oxygenation and in a higher survival rate. Moreover, early identification of HTx complications [cardiac allograft vasculopathy (CAV), primary graft dysfunction, rejection] is fundamental to improve outcomes. A variety of novel biomarkers have been explored in an attempt to identify and prevent these complications at an earlier stage. In particular, Martini et al. described the role of soluble suppression of tumorigenicity 2 (usually overexpressed in myocardial injury or cell death), donor specific antibodies (with their correlation between CAV and humoral rejection), and microRNA, as useful, albeit poorly specific, biomarkers in HTx patients.

Finally, in recent years the use of mechanical circulatory support (MCS) devices has gained ground, both as a bridge to HTx or to recovery and as destination therapy. Left ventricular assist device (LVAD) represents a valid therapeutic option in cases of patients with advanced HF who are not suitable candidates for a HTx due to associated comorbidities. LVAD can reduce mortality and improve 2-year survival significantly compared to maximal GDMT (https://doi.org/10.5603/CJ.a2021.0172). In a systematic review of Alamouti-Fard et al., it was also demonstrated that noncardiac surgeries can be performed effectively and safely on LVAD patients, providing further support for the safety of this MCS, as long as the management of these complex patients occurs at tertiary centers stuffed by healthcare providers with expertise in this field.

